# Intramedullary spinal cavernous malformations with high ossification: a case report and review of the literature

**DOI:** 10.1186/s41016-023-00323-6

**Published:** 2023-04-19

**Authors:** Weihao Liu, Chong Wang, Bo Wang, Yaowu Zhang, Wenqing Jia

**Affiliations:** Department of Neurosurgery, Beijing Tiantan Hospital, Capital Medical University, 119, South Fourth Ring West Road, Fengtai District, Beijing, China

**Keywords:** Cavernous malformation, Spinal cord, Intramedullary tumor, Calcification

## Abstract

**Background:**

Cavernous malformations of the spinal cord are a rare type of vascular malformation, comprising approximately 5 to 16% of all vascular lesions in the spinal cord. Depending on their origin position, these malformations can be distributed in different locations within the spinal canal. Although intramedullary cavernous malformations have been reported in the literature, they are exceedingly rare. Furthermore, highly calcified or ossified intramedullary cavernous spinal malformations are even rarer.

**Case presentation:**

Here, we present a case report of a 28-year-old woman diagnosed with a thoracic intramedullary cavernous malformation. The patient had been experiencing progressive numbness in her distal limbs for a period of 2 months. During routine lung computed tomography screening for COVID-19, a hyperdense mass was noted in the patient’s spinal canal. Magnetic resonance imaging revealed a mulberry-shaped intramedullary mass at the T1-2 level. The patient underwent surgical treatment, during which the entire lesion was successfully removed, resulting in a gradual improvement of her symptoms. Histological examination confirmed the presence of cavernous malformations with calcification.

**Conclusions:**

Intramedullary cavernous malformations with calcification are rare and special type that should be treated surgically in the early stage without significant neurological impairment before rebleeding or enlargement of the lesion can occur.

## Background

Cavernous malformations (CMs) are common vascular malformations of the central nervous system (CNS) and occur in about 0.16–0.4% of the general population [1, 2]. Intramedullary spinal CMs (ISCMs) are very rare and account for 5–16% of all spinal vascular malformations, which are usually solitary or may be associated with cavernous angiomas in the CNS [3]. ISCMs are primarily located in the cervical and thoracic segments. The most common clinical manifestations are sensory deficit, motor weakness, and pain [4]. Surgical resection is the preference. ISCMs with full calcification or even ossification are extremely rare. In this report, we presented a young female of ISCMs with ossification presenting with slight numbness to her toes and reviewed the available literature.

## Case presentation

A 28-year-old female presented with a 2-month history of progressive numbness in her distal limbs. The numbness initially appeared in her left toes and gradually progressed to her right toes, followed by the onset of numbness in both hands, with greater severity on the left side, and subsiding symptoms in her toes. The patient reported that symptoms worsened after physical activity and improved with rest.

**Fig. 1:**
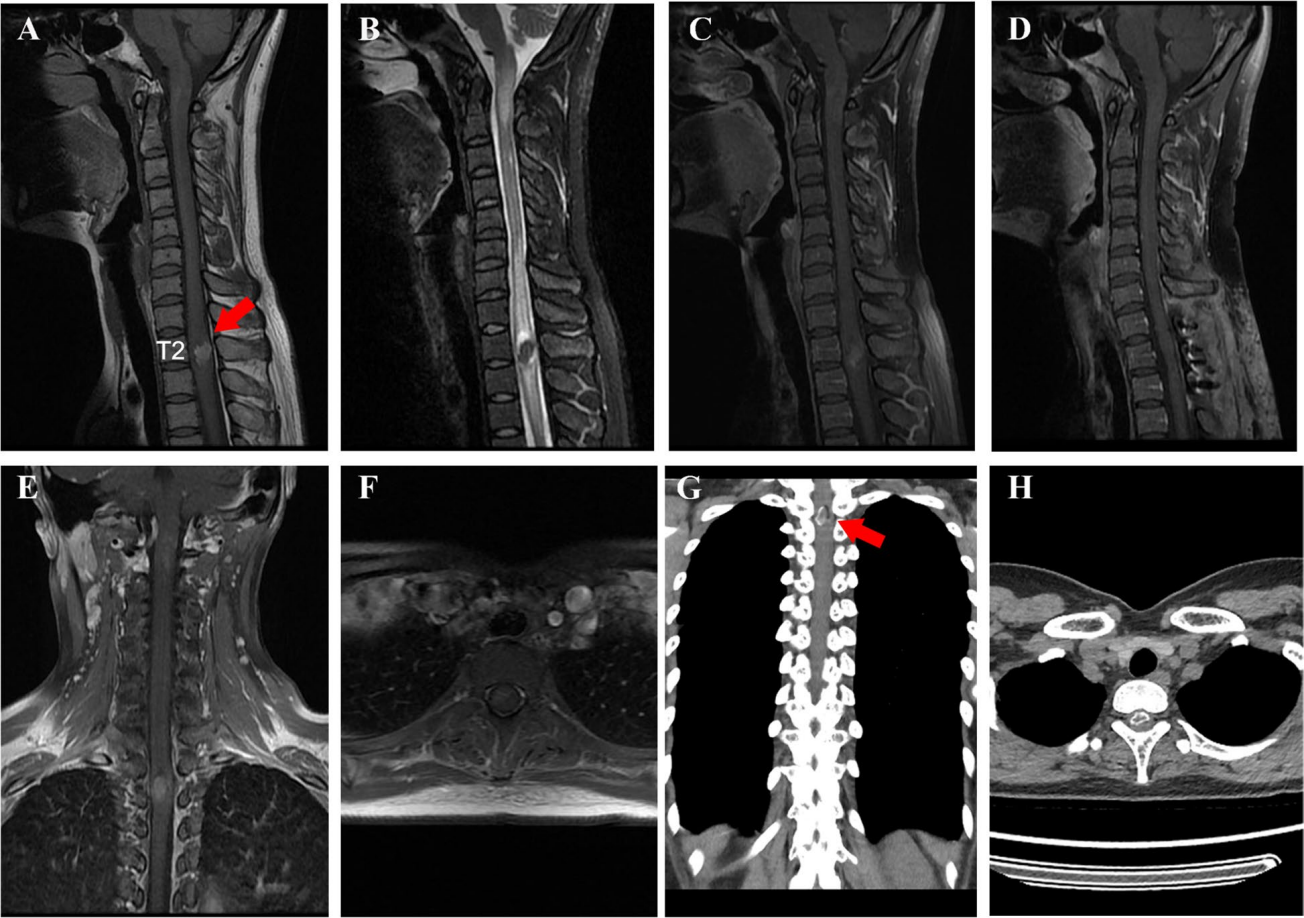
MRI and CT findings. Preoperative MRI revealed an irregular hyperintense mass in sagittal T1 (**A**) and hypointensity in T2 (**B**). The lesion was solid with hemorrhage in sagittal (**C**) and coronal (**E**) imaging after administration of gadolinium and was intramedullary on axial enhanced MRI (**F**). Postoperative contrast-enhanced MRI showed the tumor was totally resected (**D**). Preoperative CT depicted high calcification the hyperdense mass (**G**, **H**). Red arrow: location of the tumor

She was initially evaluated by the Department of Peripheral Neurosurgery and was scheduled for admission with a diagnosis of peripheral neuritis. A hyperdense mass about 15 × 12 mm^2^ was noticed in the spinal canal (Fig. 1G, H) during routine lung computed tomography (CT) screening for COVID-19. Then, the patient was transferred to the Department of Spinal Cord and Spine Neurosurgery. Physical examination revealed mild hypoesthesia below the feet. The remaining examination including strength, and cranial nerves was normal and McCormick Grade I.

Magnetic resonance imaging (MRI) showed a 15 × 12 mm^2^, mulberry-shaped intramedullary mass at the T1-2 level. T1-weighted imaging demonstrated high signal intensity, while T2-weighted imaging showed low signal intensity with spinal cord edema and no evidence of gadolinium contrast enhancement. A peripheral hyperintense lesion on T1-weighted imaging revealed subacute hemorrhage (Fig. 1A–C, E, F). This patient was diagnosed with ISCMs.

The patient underwent intramedullary tumor resection via the posterior median approach in the left lateral recumbent position under general anesthesia as well as intraoperative neurophysiological monitoring (INM) and intraoperative ultrasound.

Based on preoperative MRI and CT findings, the laminotomy and myelotomy were performed at the T1 to T3 level. The vertebra was removed using a high-speed drill, after which intraoperative ultrasound was employed to further define the tumor location and reveal a strong echogenic shadow behind the tumor.

Due to high dural tension, an incision was made in the dura and arachnoid membrane under routine microscopy, followed by a posterior midline myelotomy measuring 3 to 5 mm in size. Dorsal column mapping was utilized to minimize the potential for injury to the fasciculus cuneatus and fasciculus gracilis [5, 6].

The surface of the spinal cord is brownish yellow. The tumor was located in the ventral spinal cord of the T2 vertebral segment to the left. It was gray-red and very hard with a clear boundary, capsule, mild adhesion with surrounding tissues, and medium blood supply. The tumor envelope and the spinal cord were gradually separated along the upper pole of the tumor. The tumor in size of 1.5 × 1 × 1 cm was completely removed. The pia mater was fixed with a 7–0 PROLENE polypropylene suture (Ethicon), a duroplasty and laminoplasty, followed by a titanium microplate.

Muscle motor evoked potential (mMEP) and somatosensory evoked potential (SEP) were monitored throughout the procedure. During the tumor resection, mMEP from the left abductor hallucis disappeared and the right abductor hallucis mMEP decreased by more than 90% of the baseline amplitude.

When almost all of the tumor was removed, SEP amplitude decreased by 20% from the baseline. By the end of the procedure, SEP recovered and left mMEP was still absent. Immediately after the surgery, the patient was found with McCormick Grade III with a transient worsening of both legs. Postoperative muscle strength was grade III in the lower extremities, and methylprednisolone and hyperbaric oxygen were treated during hospitalization. On the 5th day after the surgery, the patient can stand briefly with help from others and can move her lower limbs against gravity.

On the seventh day following surgery, the patient reported acute abdominal pain, distension, and dysuria. Physical examination revealed abdominal bloating and mild hypogastrium tenderness. Abdominal ultrasound or CT of the abdomen and pelvis revealed constipation in the colon, leading to a diagnosis of fecal impaction and acute urinary retention. Treatment measures were implemented, including fasting, enema, bowel rest, intravenous rehydration, and urethral catheterization. Within 2 days, the patient’s abdominal distension significantly improved and she was discharged on the ninth day after the operation.

**Fig. 2:**
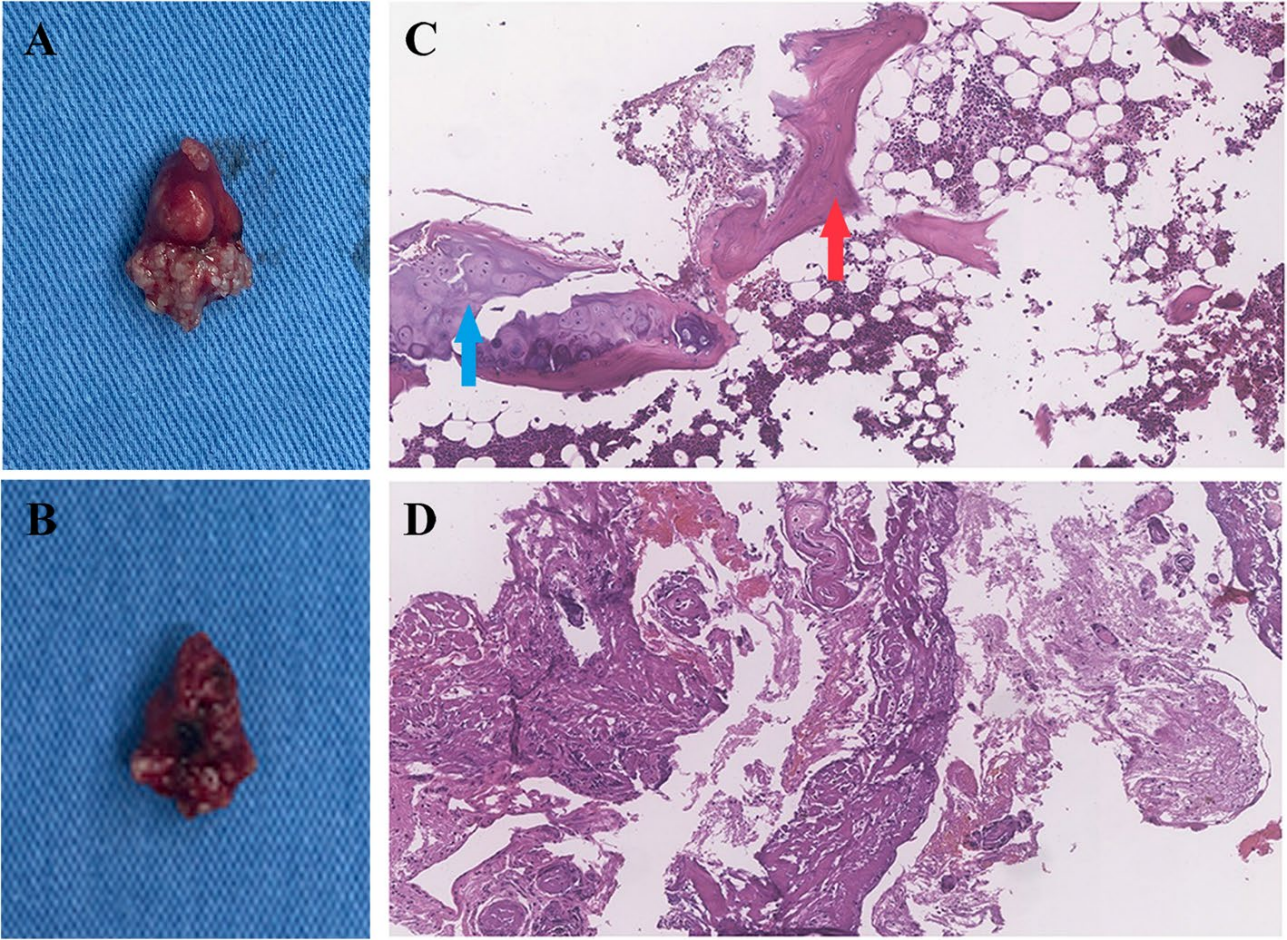
Histological and pathologic findings. An integrated mass with complete ossification on one side. Ventral (**A**) and dorsal (**B**) surfaces of the mass. Histological image (H&E, × 100) of intramedullary spinal cavernous malformations (**C**) showing the bone tissue (red arrow) and bone marrow with adipose metaplasia, marginal hyperplasia of hyaline cartilage (blue arrow). A small amount of glial tissue, collagen fibers, and blood vessels with hyaline degeneration of the vascular wall (**D**)

Postoperative gadolinium-enhanced MRI showed complete removal of the tumor (Fig. 1D). Histopathological examination confirmed the diagnosis of cavernous malformations with bone tissue and hyaline cartilage hyperplasia (Fig. 2A–D). At the 12-month follow-up, the patient had recovered her strength and her symptoms had subsided, with no signs of recurrence.

## Discussion

According to current literature, spinal cord cavernous malformations (CMs) account for 5 to 12% of all spinal vascular malformations. Studies have suggested that females are more likely to be affected than males, with a prevalence ratio of 2:1 [7–10]. Intramedullary spinal CMs with calcification or ossification are extremely rare, and only five case reports have been published on this specific pathology (Table 1). It is uncertain why calcification occurs, but it may be related to chronic arterial blood pressure, atheromatous degeneration with calcified thrombus, or immune arteritis leading to cystic necrosis of the medial artery wall. The natural history and optimal management of ISCMs remain unclear due to limited literature on the subject.

**Table 1:** Review of the 5 cases of intramedullary cavernous hemangioma with calcification of the spinal cord reported between 1985 and 2019 on PubMed

Series	Age (years) gender	Location	Presentation	Duration of symptoms (months)	Neurological function evaluation	Recurrent attacks/prior hemorrhage	Management	Histological type	Prognosis
Lin et al. [11]	18, female	T9	Acute urinary retention, limbs anesthesia, difficulty to walk	34	Lumbar level numbness, paralysis below the caudal level	Yes	Total removal	Spinal intramedullary angiomas with bone girders and bone marrow form	Lumbar level is improved, but paralysis remains below the caudal level
Tyndel et al. [12]	27, female	C6	Painful, dysesthesia of the feet	6	Right-hand mild weakness, Babinski reflex reflexes ( +)	Yes	Total removal	Non-arterial vascular channels with areas of dense calcification and hone formation	Improved, except for a slightly spastic gait,
Naim-Ur-Rahman et al. [13]	19, female	T7-8	Progressive weakness, numbness and stiffness of the legs, paraparesis, increased leg numbness, and loss of bladder control < 2 days	60	unable to stand or walk, marked weakness, and spasticity of both legs Sensation impaired in the lower extremities with a sensory level at about the umbilicus	Yes	Total removal	closely opposed vascular spaces lined by a single layer of endothelium, focal early calcification was seen	Improved, leg strength improvement and could walk with a cane after 1 year
Kang et al. [14]	61, male	T1-2	Pain in the back and both legs, progressive quadriparesis	3	Quadriparesis, weakness of both legs	N/A	Total removal	Cavernous hemangioma with calcification, ecstatic vessels with a single endothelial lining and hyalinized walls	Improved, complained mild dysesthesia on the right lower extremity
Cosgrove et al. [15]	41, female	T2-3	Gradual onset of left groin numbness, mild leg weakness bilaterally	108	Hyperreflexia with bilateral Babinski signs and ankle clonus	Yes	Biopsy of the lesion	Cavernous angioma	Unchanged for 1 year and getting worse in numbness and stiffness of the lower extremities and increased difficulty in walking

The clinical course prior to presentation was classified into five types as described by Ogilvy et al.: (1) Type A: discrete episodes of neurological deterioration with varying degrees of recovery between episodes; (2) Type B: slow progression of neurological decline; (3) Type C: acute onset of symptoms with rapid decline; (4) Type D: acute onset of mild symptoms with subsequent gradual decline lasting weeks to months; and (5) Type E: asymptomatic incidentally detected lesions [16]. In the literature review, 2 cases were type B, 2 cases were type C, and 1 case was type D. Our case was type A. In terms of surgical timing selection, this classification has a limited effect.

### Surgical indications and surgical timing

While the annual risk of bleeding in asymptomatic patients with ISCMs is low (0.8%), the cumulative risk of bleeding over time may still be significant, especially in younger patients. Therefore, surgical resection may be justified in these patients to prevent subsequent hemorrhage and neurological decline. In contrast, symptomatic patients with a history of bleeding have a higher annual risk of bleeding (9.5% and 9.7%) and may benefit more from early surgery [17].

Asymptomatic patients with ISCMs are typically managed conservatively with regular follow-up MRI scans to monitor any changes in size or symptoms. The decision to perform surgery should take into account the patient’s age, clinical status, and the size and location of the lesion [18].

Patients with ISCMs and a lesion size greater than 1 cm, presence of symptoms, and prior hemorrhage are at a higher risk of subsequent hemorrhage and neurological decline. This highlights that while oligosymptomatic patients may be managed conservatively, symptomatic patients with large lesion size greater than 1 cm may benefit from early surgery to prevent subsequent hemorrhage and neurological worsening [17].

ISCMs tend to follow a more aggressive clinical course compared to cranial cavernous malformations, and early surgical intervention with complete resection is often recommended to prevent repeated bleeding and damage to the spinal cord [4, 17, 19]. When calcification or ossification occurs in ISCMs, surgical treatment may need to be more aggressive due to the potential for increased difficulty in removing the lesion and the increased risk of complications. Based on the available information, surgery seems to be a reasonable and appropriate course of action for this patient.

### Preoperative examination

MRI remains the preferred imaging modality for ISCMs. These tumors typically exhibit mixed signal intensity on T1- and T2-weighted images. On T1-weighted images, ISCMs typically appear as isointense to hyperintense lobulated lesions with a hypointense rim. On T2-weighted images, a pathognomonic appearance of a “popcorn ball” with a complete hypointense hemosiderin rim may often be appreciated. However, rarely, ISCMs may present with a homogenous hyper or hypointense appearance. It is important to note that T2-weighted images tend to overstate the real lesion size due to the ballooning effect caused by hemosiderin deposits [20].

Hemorrhage, widening of the spine, and, rarely, calcification can also be nonspecifically appreciated on computed tomography scans. ISCMs are usually occult with digital subtraction angiography due to their low blood flow feature. MRA can better assess the blood supply and surrounding tissue of the tumor.

MRI is the preferred imaging method. The typical nuclear magnetic manifestation is ISCMs have mixed signal intensity on T1- and T2-weighted images. T1-weighted images usually demonstrate an isointense to hyperintense lobulated lesion a hypointense rim. On T2-weighted images, a pathognomonic appearance of a “popcorn ball” with a complete hypointense hemosiderin rim may often be appreciated. ISCMs may present with a homogenous hyper or hypointense appearance. Since there is a ballooning effect due to hemosiderin deposits, T2 weighted images tend to overstate the real lesion, and therefore, the T1 weighted sequence is more appropriate for the estimation of the relation between ISCMs and pial surface.

Hemorrhage, widening of the spine, and rarely calcification can be nonspecifically appreciated on computed tomography (CT) scans. Due to its low blood flow feature, ISCMs are usually occult with digital subtraction angiography. MRA can better assess the blood supply and surrounding tissue of the tumor [21].

### Surgical strategy

Meticulous presurgical planning is essential to achieve the best possible result when treating ISCMs. Although there are various approaches to the spinal cord, the posterior approach is the most commonly used. This approach has several advantages, including extensive exposure of the spinal cord, relative safety, and familiarity among neurosurgeons. However, for ventrally or ventrolaterally located ISCMs, major myelotomy and spinal cord traction should be avoided, and other surgical approaches should be considered, such as the anterior, anterolateral, transthoracic, or posterolateral approach [22, 23].

There are three possibilities for bone removal during surgery: laminectomy, laminoplasty, and hemilaminectomy. We believe that laminoplasty provides better intraoperative visual field and exposure, as well as spinal stability. Therefore, it is often the preferred method for bone removal in the treatment of ISCMs.

It is important to note that the three recommended non-eloquent entry zones in the spinal cord for accessing ISCMs are the dorsal root entry zone (DREZ), dorsal median sulcus, and lateral entry zone located between the ventral and dorsal nerve roots [24].

In this case, where the ISCMs are deep-seated and calcified, a midline myelotomy is often necessary. Electrophysiological monitoring, including D-wave and point reversal techniques, is necessary to ensure the safety of the procedure [25]. The dissection plane should be followed between the ISCMs and spinal cord, leaving the gliotic tissue at the periphery in place. It is recommended to remove ISCMs en bloc, and debulking using a Cavitron Ultrasonic Surgical Aspirator is generally not recommended. In highly vascular tumors, near-infrared indocyanine green video angiography can provide real-time information about vascular flow dynamics during the surgery, and it can help surgeons localize the normal circulation of the spinal cord, as well as the feeding arteries and draining veins [26]. After resection and meticulous cauterization, the pia mater should be fixed with a 7–0 PROLENE polypropylene suture, and a watertight suture of the dura should be performed. High doses of hormones such as 500 mg of methylprednisolone and mannitol may be used during the operation to reduce spinal tone.

### Postoperative rehabilitation

In the reported case, the ISCMs occupying space and subacute bleeding resulted in spinal cord impairment. Postoperative rehabilitation programs are essential for optimizing recovery and can include hormonal therapy, hyperbaric oxygen therapy, and exercise rehabilitation [27]. Surgical treatment by a fully evaluated and skilled surgeon can eliminate the risk of new bleeding from ISCMs and has a favorable prognosis for recovery.

## Conclusions

Since ISCMs are rare, our case provides a new subset for studying the tumor growth pattern of ISCMs and we believe that early standardized surgical treatment can benefit patients.

## Data Availability

Not applicable.

## Abbreviations

CMs: Cavernous malformations
CNS: Central nervous system
ISCMs: Intramedullary spinal CMs
CT: Lung computed tomography
MRI: Magnetic resonance imaging
INM: Intraoperative neurophysiological monitoring
mMEP: Muscle motor evoked potential
SEP: Somatosensory evoked potential
